# Common aquatic pollutants modify hemocyte immune responses in *Biomphalaria glabrata*

**DOI:** 10.3389/fimmu.2022.839746

**Published:** 2022-09-08

**Authors:** Adam E. Lynch, Leslie R. Noble, Catherine S. Jones, Edwin J. Routledge

**Affiliations:** ^1^ College of Health, Medicine and Life Sciences, Brunel University London, Uxbridge, United Kingdom; ^2^ Faculty of Biosciences and Aquaculture, Nord University, Bodø, Norway; ^3^ School of Biological Sciences, Aberdeen University, Aberdeen, United Kingdom

**Keywords:** pollution, motility, encapsulation, phagocytosis, gastropoda, immune effector cells, immunomodulation

## Abstract

Disruptions to reproductive health in wildlife species inhabiting polluted environments is often found to occur alongside compromised immunity. However, research on impacts of aquatic pollution on freshwater mollusc immune responses is limited despite their importance as vectors of disease (Schistosomiasis) in humans, cattle and wild mammals. We developed an *in vitro* ‘tool-kit’ of well-characterized quantitative immune tests using *Biomphalaria glabrata* hemocytes. We exposed hemocytes to environmentally-relevant concentrations of common aquatic pollutants (17β-estradiol, Bisphenol-A and p,p’-DDE) and measured key innate immune responses including motility, phagocytosis and encapsulation. Additionally, we tested an extract of a typical domestic tertiary treated effluent as representative of a ‘real-world’ mixture of chemicals. Encapsulation responses were stimulated by p,p’-DDE at low doses but were suppressed at higher doses. Concentrations of BPA (above 200 ng/L) and p,p’-DDE (above 500 ng/L) significantly inhibited phagocytosis compared to controls, whilst hemocyte motility was reduced by all test chemicals and the effluent extract in a dose-dependent manner. All responses occurred at chemical concentrations considered to be below the cytotoxic thresholds of hemocytes. This is the first time a suite of *in vitro* tests has been developed specifically in *B. glabrata* with the purpose of investigating the impacts of chemical pollutants and an effluent extract on immunity. Our findings indicate that common aquatic pollutants alter innate immune responses in *B. glabrata*, suggesting that pollutants may be a critical, yet overlooked, factor impacting disease by modulating the dynamics of parasite transmission between molluscs and humans.

## Introduction

Schistosomiasis is a parasitic disease, transmitted *via* tropical freshwater snails such as *Biomphalaria glabrata*, afflicting more than 200 million people in 70 countries, killing over 250,000 annually in sub-Saharan Africa alone. This public health challenge impacts negatively on the economy and welfare of many Lower to Middle Income Countries (LMICs) throughout Africa, South America, the Caribbean, the Middle East, and Asia. Transmission relies on infective fecal matter or urine carrying schistosome eggs entering freshwater, whereupon a ciliated mobile phase (miracidium) hatches, seeking and infecting particular species of tropical freshwater snails (the intermediate host). In order for schistosomes to complete their life cycle, the miracidia must initially burrow through the skin of the appropriate snail species, evading its immune defenses to transform and reproduce asexually, amplifying many thousand fold into the human infective form (cercaria). Rural populations depend on rivers for recreation and livelihoods, which combined with poor sanitation infrastructure propagates a cycle of infection and reinfection. The parasite can remain in the body for years, with eggs damaging vital organs such as the bladder, kidneys and liver, often triggering immunopathologies. The drug Praziquantel kills adult worms but does not prevent reinfection. Consequently, schistosomiasis is both a cause and a consequence of poverty in many LMICs.

Parasite-host interactions can help elucidate two essential life processes; reproduction and immunity. Although often considered separate systems in intact organisms, they are in fact intricately connected through shared biological pathways. Research in vertebrates has revealed that steroid estrogens (critical signaling molecules in reproduction) affect major cellular components of the immune system (1), including T, B and antigen-presenting cells. In fact, estrogens mediate crosstalk between vertebrate reproductive and immune systems by orchestrating the expression of various cytokines including interferon-gamma and proinflammatory cytokines such as Interleukin 4 and 6, and Tumour Necrosis Factor-alpha (see (2)). The expression of the estrogen receptor (ERα), and its constitutively active ligand-independent form (the estrogen related receptor alpha; ERRα), in activated mouse macrophages highlights the importance of estrogen receptor-mediated signaling pathways in vertebrate innate immune function (3), and explains its susceptibility to interference by chemical pollutants, including endocrine disrupting chemicals (EDCs) with estrogenic and anti-estrogenic properties (4). However, EDC targets of the immune system will not be confined to estrogen agonists and antagonists alone, for the glucocorticoid-receptor (GR), Liver X Receptors (LXR) and Peroxisome Proliferator Activated Receptor gamma (PPARγ) present in macrophages regulate both innate and acquired immune responses (5). EDCs may also affect immunity *via* non-genomic mechanisms (such as inhibiting cytochrome P450 steroid biosynthesis) or by altering metabolism (6). It is perhaps not surprising then that polluted environments (contaminated with EDCs such as PCBs and DDT) are associated with both altered reproduction and compromised immune function in a variety of wildlife species, including birds (7, 8), reptiles (9, 10), fish (11) and marine mammals (12, 13).

Although vertebrates have the most sophisticated immune systems, immune capacity exists in all animal phyla, from protozoans, to marine tunicates and invertebrates. It is generally agreed that invertebrates possess only an innate immune response, which nonetheless demonstrates immune memory (see 14), whereas vertebrates possess both innate and adaptive immunity. The innate system is often referred to as ‘basic’ immunity, though this term does not imply inferior or superseded immunity, since invertebrates make up 95% of all animal species (15–17). Most invertebrates have an open circulatory system in which a fluid termed ‘hemolymph’ is contained in the body cavity or ‘hemocoel’ and directly bathes the organs (18, 19). The hemolymph, similar in function to vertebrate plasma, contains immune components including antimicrobial peptides, agglutinins and lysosomal enzymes. These enhance opsonization by facilitating bacterial aggregation and immobilization and/or display cytolytic activities (20). Free-floating in the hemolymph are the principal effectors of the invertebrate immune response, ‘hemocytes’ (20). Analogous to vertebrate macrophages, a vital function of hemocytes is the process of phagocytosis of small foreign particles (21). The phagocytic process has a sequence of distinct stages: attraction (chemotaxis), attachment, engulfment and digestion; an effective evolutionary conserved process (22, 23).

Recent genomic analysis has shown *B. glabrata* displays a ‘multifaceted, complex internal defence system that must be evaded or negated if parasites such as *S. mansoni* are to successfully establish an infection’ (24). This includes a wide variety of pattern recognition receptors (PRRs), cytokines, as well as gene orthologues of complement factors (24). Molluscs, including *B. glabrata*, also express both ERα and ERR orthologues (25), although their functional role in mollusc physiology is unclear. Sexually dimorphic patterns of expression [i.e. relatively high levels of mcER-like in the penis and sheath, and comparatively low expression of mcERR in female accessory sex tissues in *M. cornuarietis* (26)], suggest they are unlikely to be redundant. The notion that steroid hormones recognized in vertebrates are necessary for mollusc reproduction is widely debated in the literature (27), because *de novo* synthesis is unproven, and the mollusc ERα orthologue neither binds to, nor is it activated by, vertebrate steroid estrogens (28). Despite this, exposure to 17β-estradiol and some xenoestrogens (e.g. Bisphenol A, DDT) are reported to affect reproduction in various mollusc species (29, 30). This is important considering the natural environment is subject to systematically increasing concentrations of chemical pollutants, including xenoestrogens (31–33). Although 17β-estradiol reportedly affects immune responses in hemocytes of the mussel *Mytilus galloprovincialis* (34), the role of the ER in mediating mollusc immune responses remains unknown. *In vitro* evidence, however, suggests that Bisphenol A (widely reported to be a weak ER agonist), interacts with the ERR in both molluscs and humans (28, 35), is a thyroid receptor antagonist (36) and an androgen receptor antagonist (37). The possibility exists that BPA may also exert pro-inflammatory outcomes by virtue of antagonizing an important PPAR regulatory/anti-inflammatory axis [reviewed in (38)]. Therefore, chemical pollutants, including many EDCs, may have the capacity to interfere with signaling pathways shared by a variety of processes in molluscs, including metabolism, immunity and reproduction, just as they do in vertebrates.

In the absence of (i) published, well-characterized and quantitative immune tests in gastropod molluscs for toxicology testing purposes, and (ii) a comprehensive understanding of immunomodulatory effects of chemicals on molluscs, our goal was to develop a ‘tool-kit’ of *in vitro* immune tests using mollusc hemocytes to assess whether common aquatic pollutants can interfere with the immune system of the freshwater gastropod *B. glabrata*. To achieve this, we extracted hemocytes from *B. glabrata* to investigate whether important immune processes, such as motility, phagocytosis and encapsulation could be altered when challenged with different concentrations of common aquatic pollutants (including the steroid 17β-estradiol, the plasticizer Bisphenol-A and the main metabolite of the pesticide DDT) due to their reported presence in active concentrations in the aquatic environment, variation in the possible modes of action relevant to mollusc immune function, and their association with reproductive and immune effects in both vertebrates and invertebrates. As pollutants do not exist in nature in isolation, we also tested an effluent extract as a representative real-world mixture of chemicals. The choice of *B. glabrata* is significant as it serves as the intermediate host of *Schistosoma mansoni*; a trematode parasite responsible for a debilitating neglected tropical disease in humans, second only to malaria as the most devastating parasitic disease in terms of socioeconomic importance and public health impact (39). This is the first time that a suite of *in vitro* tests capturing critical immune responses have been developed in *B. glabrata* to address the impacts of chemical pollutants and an effluent extract on hemocyte immune function.

## Materials and methods

### Biomphalaria husbandry

A stock of *B. glabrata* (strain BB02 - sourced from the Natural History Museum (London)) were reared at a density not exceeding 10 snails per liter in a flow-through system fed with RO water reconstituted with minerals and salts (to prevent the snails developing fragile shells). Photoperiod was maintained at 12h light/dark, and water temperature between 24-28°C using submersible aquarium heaters. Snails were fed three times a week with commercial fish flakes (TetraMin^®^) tested previously for estrogenic activity and known to be free of known estrogenic chemicals which may affect physiological responses in animals. At each feeding event the quantity of food was limited to the amount that the snails could consume within a single day, and tanks were cleaned regularly to maintain appropriate water quality parameters.

### Biomphalaria cell culture

Molluscan hemocytes are a notoriously difficult cell type to manipulate *in vitro*, due to their propensity to clump together, often irreversibly after centrifugation, and to adhere strongly to various substrate including collection vessels (40). Specialized techniques have been developed specifically to permit their use *in vitro*. Chernin’s balanced salt solution (‘CBSS’; (41)) was designed to mimic the salt composition, osmolarity and pH of the hemolymph. Hemocytes behave normally in CBSS and display phagocytosis and encapsulation even in the absence of hemolymph (24, 42). CBSS was the primary buffer used for *in vitro* maintenance and manipulation of hemocytes, and as a carrier for chemicals or other materials that hemocytes were exposed to. In order to collect, recover and fix/analyze hemocytes after completion of exposures to chemicals, modified buffers containing chelating agents (α-CE; EDTA, α-CC; caffeine) were used. These buffers maximize detachment or survival, by rounding-up cells which have spread on glass and reduce clumping during low speed centrifugation. A detailed composition of the buffers is reported in (40).

### Hemolymph collection

The following protocol (adapted from (43) was used to obtain a pooled sample of sterile hemolymph for *in vitro* experiments. Snails (~4mm shell length) were collected and the shells dried with paper towel and then carefully swabbed using cotton buds soaked in 70% ethanol, taking particular care to clean inside the shell whorl. After air drying on paper towel for 1-2 minutes inside a laminar flow cabinet, snails were then placed in an autoclaved beaker containing sterile filtered water (maximum of 18 snails per 100ml) plus 2% antibiotic-antimycotic solution (10,000 units/ml penicillin, 10 mg/ml streptomycin and 25 ptg/ml amphotericin B; Sigma-Aldrich, UK) and left for 45 minutes at 27°C. After this time, snails were carefully removed from the beaker using sterile forceps and placed on a fresh paper towel inside the cabinet to dry for 1-2 minutes. The shells were again dried with paper towel, swabbed with 70% ethanol and the snail placed in an autoclaved glass petri dish. After the ethanol had evaporated (within 30 seconds) hemolymph (approximately 300-400ul/snail) was extracted with a sterile scalpel according to the ‘headfoot puncture protocol’ described by (44). Hemolymph was allowed to collect for only a short period of time (< 5 minutes) to avoid cells adhering to the dish. Hemolymph from each snail was then removed from the dish using a sterile p200 pipette and combined into a single falcon tube on ice (to prevent clumping) through a 70μm cell strainer (Falcon^®^ Cell Strainer; Fisher Scientific Co., UK) to prevent shell debris or cell clumps passing into the sample. The research was conducted with ‘Animal Research: Reporting *In Vivo* Experiments’ (ARRIVE) guidelines (45) in mind.

### Preparation of chemical stocks

Master stock solutions of 17β-estradiol (2.5g/l >99.9% pure), Bisphenol-A (200mg/L ≥ 99%) and p,p’-DDE (500mg/L ≥98%) dissolved in analytical grade ethanol (Sigma-Aldrich, UK) were prepared in solvent-rinsed clear glass sample vials with rubber-lined screw cap lids. Master stock solutions were serially diluted in 10-fold steps to produce the concentration range of working stocks needed for the various immune assays. Ethanol containing chemicals was evaporated to dryness before dissolving in CBSS whenever possible. 

### Preparation of the effluent extract

Six 47mm Octadecyl C18 extraction disks (EmporeTM) layered with 1cm of Filteraid glass beads (~50μm, Empore) were secured into the extraction manifold. Each disk was conditioned and primed using a sequence of solvent washes (methanol, ddH2O and methanol) according to the manufacturer’s instructions. Treated domestic effluent was then drawn through each disk under vacuum. Disks required changing every 1-3L due to gradual blocking resulting in substantial decreases in filtering rate. Following extraction, used disks were left to air dry under vacuum for ~30 minutes. The pump was then turned off and the glass apparatus (as well as the disk) was removed from the manifold in one piece with the clamp in place. The outlet of each manifold was placed inside a separate glass collection tube (15ml) and positioned to avoid obstructing the air flow. 10ml of methanol was then allowed to pass slowly through the disk and into the collection tube to ensure maximum recovery of compounds from the disk. Tubes containing the concentrated effluent extract in 100% methanol were then sealed with screw-tops and Parafilm and stored at 4°C. The extracts were then evaporated to dryness using a TurboVap operating at 50°C under nitrogen (~15 bar). Samples were periodically checked until they reached ‘incipient dryness’; the point at which the methanol had essentially evaporated with only enough remaining to leave a viscous residue (rather than a completely dry sample) that could be more easily dissolved in ethanol. Finally, 1ml of ethanol was added to each centrifuge tube, the tubes were mixed vigorously until no obvious residue remained. Concentrated extracts were then pooled together to form the final stock with a known concentration relative to the original sample.

### Incubation of hemocytes with test chemicals and effluent extract

All test chemicals and the effluent extract were made up in analytical grade ethanol (Sigma-Aldrich, UK). Controls (i.e. those containing no test-chemical) contained an amount of ethanol equal to the chemical doses to account for possible solvent effects. Chemical stocks were serially diluted in absolute ethanol to produce a range of working stocks, and these were aliquoted into the relevant well, allowed to evaporate to dryness and then dissolved in CBSS. In order to avoid excessive manipulation of hemocytes, and the associated problems with cell clumping, the desired concentration of test chemical in the hemocyte sample was achieved by making a double strength (2x) solution in CBSS and then mixing this with an equal volume of hemolymph to produce a solution of 50% hemolymph and 50% CBSS/test chemical.

### Parasite culture

As *in vitro* assays required working with snail-infective stages of *S. mansoni* it was necessary to harvest eggs from infected tissues, hatch the eggs to produce miracidia, and transform these into sporocysts. The sporocyst stage is representative of the earliest stage of the parasite in snail tissue, and is the form that is subject to initial attack from the snail immune system.

#### Mouse stage and egg collection

All parasites were obtained by infection of mice carried out under the United Kingdom Animal’s Scientific Procedures Act 1986, at the London School of Hygiene and Tropical Medicine under their project license. Mice (purchased from Charles River, Margate, Kent, UK) were typically infected by subcutaneous injection of approximately 100 cercariae. After 7-8 weeks (or sooner if visibly suffering from the infection), animals were euthanized with an intra-peritoneal injection of Tiletamine/Zolazepam (800 mg/kg) and Xylazine (100 mg/kg). Following euthanasia, the adult worms were collected from mice by perfusion of the hepatic portal system and the eggs collected by removal of the liver.

#### Egg extraction and hatching

Eggs were typically harvested from infected mouse livers within 24h of removal to ensure optimum yields. The infected mouse liver can be stored at 4°C for 48 to 72 hours, but recovery of viable miracidia reduces steadily over time. The liver was first washed with a solution of 1.2% NaCl to remove excess tissue and then placed on a metal sieve with a 180 μm pore mesh. The sieve was placed on top of a conical measuring flask. The tissue was manually homogenized using a porcelain pestle, with the addition of more NaCl solution to aide passage through the mesh. Once all the tissue had passed through the sieve the flask was topped up with 1.2% NaCl to a final volume of 200ml, at which point it was placed in a fridge at 4°C for 30 minutes. The combination of saline solution and low temperature prevents premature hatching of the eggs. After this settling period a tissue sediment was formed, the flask was removed and half of the supernatant was carefully poured off and discarded, ensuring minimal disturbance of the sediment. The flask was then topped up to 200ml with fresh 1.2% NaCl and returned to 4°C for a further 30 minutes. This process was repeated 2-3 times until the supernatant appeared clear at which point the majority of the solution was poured away (leaving the sediment undisturbed) and replaced with freshwater.

#### Miracidia collection and transformation into sporocysts

The method was adapted from (46) which uses the positive geo and phototaxic responses of miracidia to separate themselves from settled tissue debris resulting in a clean parasite sample for immuno-histochemistry, molecular, and biochemical studies. The system essentially uses a wooden box fully enclosing a 500ml conical filtering flask with side arm (**Figure S1**). The side arm is accessible through a small hole on one side only, just large enough to attach a collection jar externally to the box onto the side arm of the flask which is exposed to light. The side arm was also modified by inserting a p20 pipette tip (secured by a non-toxic silicone) to serve as a funnel trap. This allows miracidia to pass into the collection bottle by swimming through the wide end, whilst preventing their return into the flask through the narrow end on the tip. The inside of the box was painted black and a foam seal was placed around the hole to reduce the chances of stray light entering the main flask. Although a small amount of light inevitably entered the main flask through the collection jar this was advantageous as it formed a phototaxic gradient without which the parasites might be unable to locate the main light source and enter the collection jar. Transformation media was added to the collection jar to further encourage the movement of miracidia towards the collection jar and to cause their transformation into sporocysts. Undiluted transformation media causes miracidia to immediately cease movement and transform into sporocysts, thereby reducing the likelihood of miracidia escaping back into the main flask and also generating the life stage needed for *in vitro* tests. To collect parasites, the cleaned egg/liver homogenate solution was poured into the main flask and allowed to settle for 30 minutes, after which the collection jar (containing transformation buffer) was exposed to light. The flask was placed at an angle of 15 degrees in order to encourage movement of transformation media from the collection chamber into the flask, thereby producing a geotaxic gradient for miracidia to follow (**Figure S2**). Once miracidia had ceased entering the collection jar, it was unscrewed from the side-arm jar and sealed with an unmodified lid. Parasites were then transferred to well-plates and left to complete transformation into sporocysts. Due to the 6-8 week intervals in the delivery cycle of eggs harvested from mouse livers sourced at LSHTM, it was decided to fix transformed sporocysts with Karnovsky’s fixative (2.5% gluteraldehyde plus 2.5% formaldehyde in CBSS buffer) for use in encapsulation assays.

### Encapsulation assay

Sporocysts in transformation buffer were removed from the culture dishes into a 15ml falcon tube containing 10ml CBSS. The tube was placed on ice to reduce further development and allow the sporocysts to naturally settle to the bottom of the tube over 15 minutes. Supernatant was extracted and replaced with Karnovsky’s fixative solution at room temperature. Once fixed, the concentration and purity of the sporocyst ‘stock’ was assessed under a microscope. Fixed sporocysts could be used after 1 hour of fixing or after considerably longer periods of time. After fixing, the required number of sporocysts were stained in the dark with 5μg/ml solution FITC (Fluorescein isothiocyanate made up in CBSS) for 25 minutes to aid identification of fully encapsulated sporocysts from dispersed cell aggregates in the assay (47) as illustrated in the Supplementary Information. Staining was adapted from the protocol used by (48). After the incubation period a washing step was performed whereby the sporocysts were spun at 2000rpm for 5 minutes and the supernatant removed and replaced with an equal volume of FITC-free CBSS. This process was repeated approximately three times until the CBSS was free of visible stain and the tube was gently vortexed to separate the sporocysts. A simple chamber slide (1cm^3^) was developed using a siliconized Vacutainer tube and non-toxic silicone putty (**Figure S3**). Chamber slides materials were sterilized by soaking in 70% ethanol and allowed to dry under a cell-culture hood. The components were assembled under the hood and the slide was then subject to UV for 30 minutes. Hemolymph was collected from snails, and three separate pools were made, each containing equal amounts of hemolymph from eight snails and placed on ice (see **Figure S6** for approach). For each separate slide/chemical dose a sample of 200μl hemolymph was taken from each pool, mixed with an equal volume of 2x (double-strength) chemical in CBSS and added to the chamber slide (n=3 for each dose). Stained sporocysts were added to the chamber slide at a ratio of 2 per μl of hemolymph, and each chamber was then sealed with a vacutainer top to prevent desiccation and incubated at 27°C for 4 hours. After the incubation period most of the liquid was removed leaving only approximately 50μl, along with the settled stained sporocysts and hemocytes. The chamber wall was then removed, and a cover-slip was placed over the sample and the slide then taken for counting under a fluorescence microscope. Analysis was undertaken using the index used by (49) illustrated in **Figure S4** and **Figure S5**. All sporocysts on the slide were counted and assigned values of 1 (no cells attached), 2 (up to 10 cells attached), 3 (>10 cells, <50% coverage) or 4 (>50% coverage). The individual values were tallied to give a total score which was then divided by the total number of sporocysts counted, to produce the final encapsulation index value. The relationship between dose (X) and encapsulation index score (Y) was modelled by first, second or third order polynomial regression models. All values for Y were entered as three independent observations (n =3) and values were not averaged prior to analysis, nor were they transformed. X values were log transformed.

### Motility assay

Cell motility tracking was performed on *B. glabrata* hemocytes using a low-cost motility tracking system (LOCOMOTIS) reported previously (50). Sterile *B. glabrata* hemolymph was collected as described previously and cells were diluted 50% in snail saline (CBSS PH 7.4; (41)) and allowed to settle for 30 minutes at 27°C before time-lapse began. *B. glabrata* hemocytes were left to attach to the plate or were placed onto a surface of 0.01% poly-L-lysine according to (51) with some minor modifications. All time-lapse images were taken every minute for 1 hour. For each chemical and dose, results were collected from four independent assays (cell pools) on different days (see **Figure S6** for further information). Analysis was performed on the MTrack2 data generated from the time-lapse files. A Kruskal-Wallis test was performed in SPSS version 20 (IMB) to determine whether recorded velocity values differed significantly for the various exposure treatments. As a *post-hoc* test, pairwise comparisons were performed using Dunn’s procedure with a Bonferroni correction for multiple comparisons. The dose-response curves for the test chemicals were plotted in Graphpad Prism 6.0 using linear and non-linear regression.

### Phagocytosis assay

Cells were prepared as a monolayer and were exposed to the same chemicals at the same concentrations as previously described. 10ug/ml cytochalasin B in CBSS was used as a control since it has been shown to be a potent inhibitor of phagocytosis in mollusc hemocytes (52). All other parameters were the same as those used for the chemical exposure study. 400μl of pooled hemolymph was added to individual wells of a 24-well plate and the cells were allowed to adhere for 30 mins at 27°C. After 30 mins the supernatant (including non-attached cells) was removed and replaced with 400μl of test chemical in CBSS and incubated for a further 30 mins at 27°C. After pre-incubation with chemicals or solvent control, 10ul of a 1/10 diluted stock of 1.0um green amine modified latex beads (Sigma, L2778) as antigens was added, and the plates were incubated at 27°C on a gentle shaker for 2 hours. Latex beads provided a relatively uniform size and high degree of fluorescence (53) for visualization. Following incubation, the supernatant was then aspirated and replaced with 200μl of α-CE to detach the cells, and transferred to an Eppendorf and kept on ice until use in the ImageStream. Imaging flow cytometry (IFC) was performed using the ImageStreamX system (Amnis Inc., Seattle, Washington). The 40x objective was used to acquire images in each experiment.

ImageStream settings were controlled using the INSPIRE software interface to analyze 3000 cells from four independent replicates (pools) for each chemical dose. Classifiers (used to determine single cells and exclude cell clumps and debris according to size) and laser power (to visualize beads correctly) were set at: 75-300, 488nm laser excitation: 20, 785nm laser excitation to exclude debris (e.g. cell clumps). Based on the population of cells in focus, a scatter graph of area vs. aspect ratio was produced using IDEAS 6.0 software, and a region drawn to define single cells while excluding clumps, non-cellular debris and free (i.e. non-internalized) antigens post data acquisition informed by individual image data collected from the ImageStream. To exclude cells in which the beads were not internalized, a morphology mask corresponding to the inside of the cell was created based on the bead positive population. To quantify the number of internalized beads a spot count feature based on the internalization positive population was created using the morphology/spot count mask. This function counts objects with high fluorescence which fall within the mask thereby providing a count of the number of beads inside each internalization positive cell.

All dose-response data collected from the ImageStream was subsequently analyzed using non-linear regression. Number of beads per cell was treated as continuous in order to utilize the spot count data.

### Validation experiments

The following validatory tests were performed to help ensure that sub-lethal immune effects of chemicals on hemocytes were being measured, and to avoid potential confounders, such as direct cytotoxicity.

#### Dose-range effects of test chemicals on hemocyte viability

For each replicate, 100μl pooled hemolymph was collected as described earlier and mixed with an equal volume of CBSS, containing the dose range of test chemical, and transferred to wells of a 24-well plate. After incubation for 1-hour at 27°C, the majority of hemocytes had spread to form a monolayer. The supernatant was removed from the monolayer and replaced with 100μl α-CC and incubated for a further 10 minutes. After the final incubation an equal volume of 0.4% trypan blue was added to the well and the solution mixed. Immediately after mixing, a 10μl sample was taken and added to a hemocytometer where 100 cells were assessed for exclusion or incorporation of the dye. This process was repeated three times for each experimental replicate of each dose (see **Figure S6**).

#### Inhibitor (sodium azide) dose-response for *in vitro* assays

To determine if chemically-induced alterations in the immune-response of interest can reliably be measured, it was important to include compounds with established and reliable effects which are not due to direct cytotoxicity. Sodium azide (NaN3) is known to be a potent cytochrome C inhibitor in many different cell-types from numerous species, including molluscan hemocytes (54). Inhibition of cytochrome C results in the inhibition of ATP-synthesis, ATP being the main source of energy for the majority of cellular functions (55). Cellular functions dependent on ATP, and thus shown to be inhibited by sodium azide, include phagocytosis, motility and aggregation (56) (54). In studies involving molluscan hemocytes sodium azide, at concentrations of up to 2%, has been shown to considerably reduce phagocytosis without significant reduction in cell viability (57, 58).

## Results

### Dose-response of individual chemicals and effluent extract on hemocyte viability

**Figure 1:**
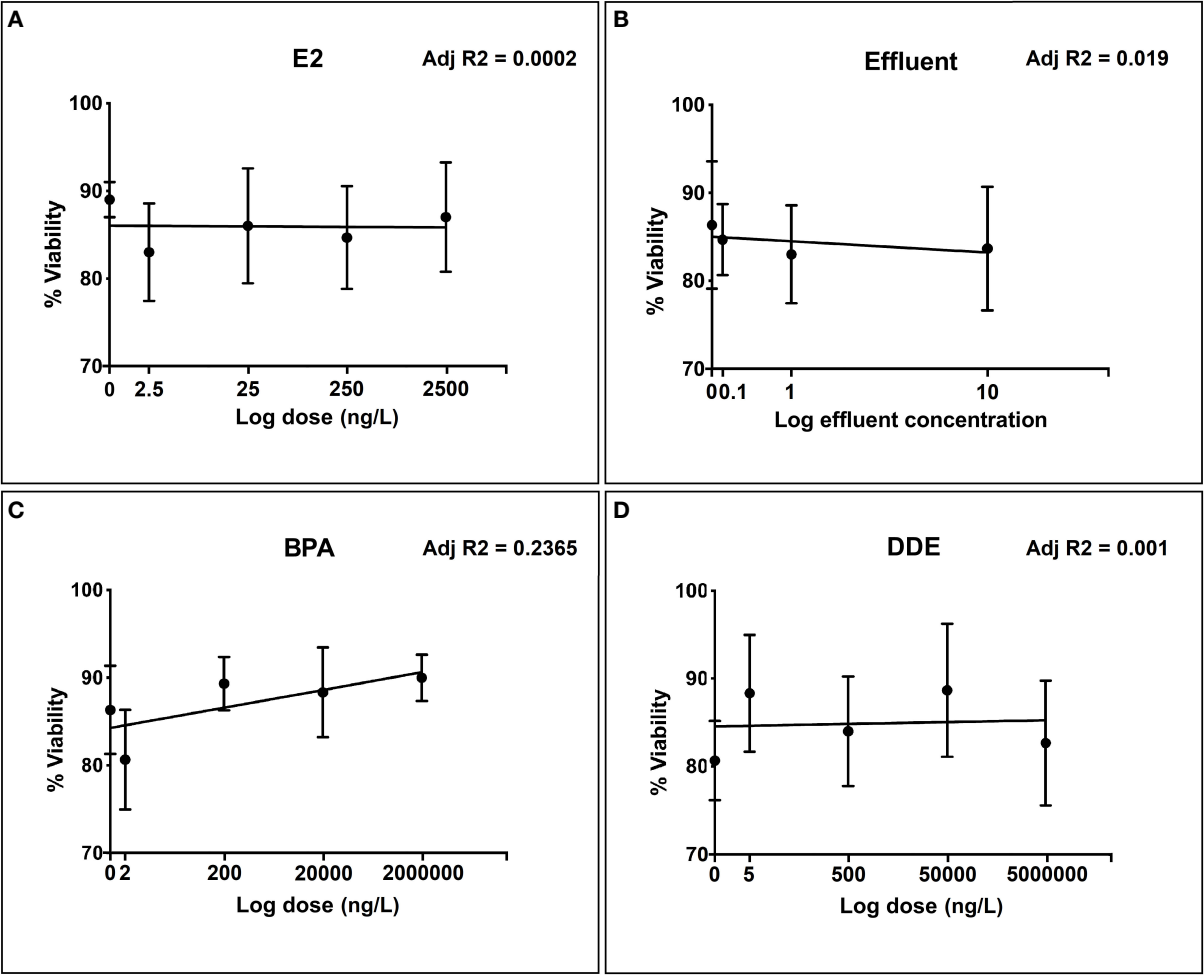
Toxicity evaluation of different doses of selected chemicals to snail hemocytes over the selected dose range for each test chemical. **(A)** 17β-estradiol (E2); **(B)** Effluent extract; **(C)** Bisphenol-A (BPA); **(D)** p’p-Dichlorodiphenyldichloroethylene (p,p’-DDE). Hemocyte viability measured as % of viable cells (± SEM of three independent replicates) according to Trypan blue exclusion.

Percentage viability in unexposed (control) hemocytes typically ranged between 80% and 90%. None of the test chemicals showed any meaningful relationship between increase in dose, within the concentration range, and reduction in cell viability according to the trypan blue exclusion assay (**Figures 1A** **–D**). There was a very slight positive relationship between viability and exposure concentration for BPA (**Figure 1C**), which was probably due to natural variability and low viability in the lowest exposure group.

### Effect of sodium azide (NaN3) inhibitor on the performance of the different immune assays

**Figure 2:**
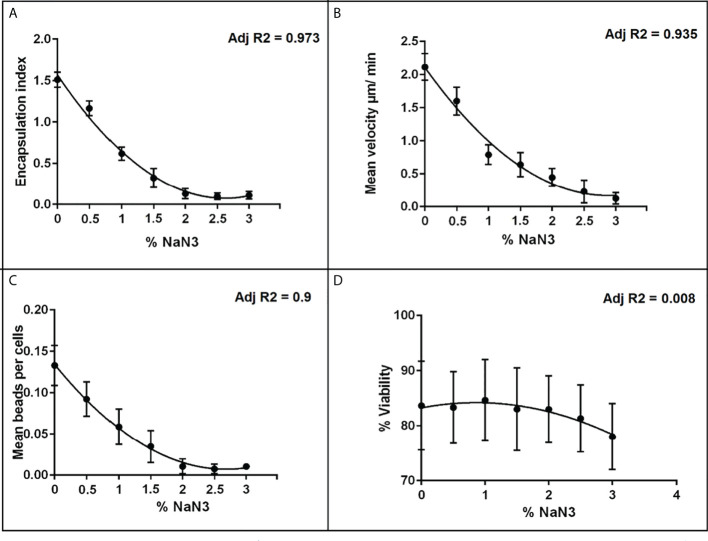
Dose-response relationships between the metabolic inhibitor NaN3 and different hemocyte immune endpoints. **(A)** encapsulation index; **(B)** mean velocity; **(C)** phagocytosis and **(D)** % viability, plotted points represent the SEM of three independent replicates. Adj R^2^ is the regression value based on the mean response at each dose.

**Figures 2A–C** shows that the response of each immune-endpoint was strongly suppressed with increasing doses of NaN3, while hemocyte viability was minimally affected across the same dose range (**Figure 2D**). These results are in accordance with previous reports on the ability of NaN3 to suppress hemocyte function at similar concentrations, without inducing significant loss in viability (54, 58).

### Encapsulation assay

**Figure 3:**
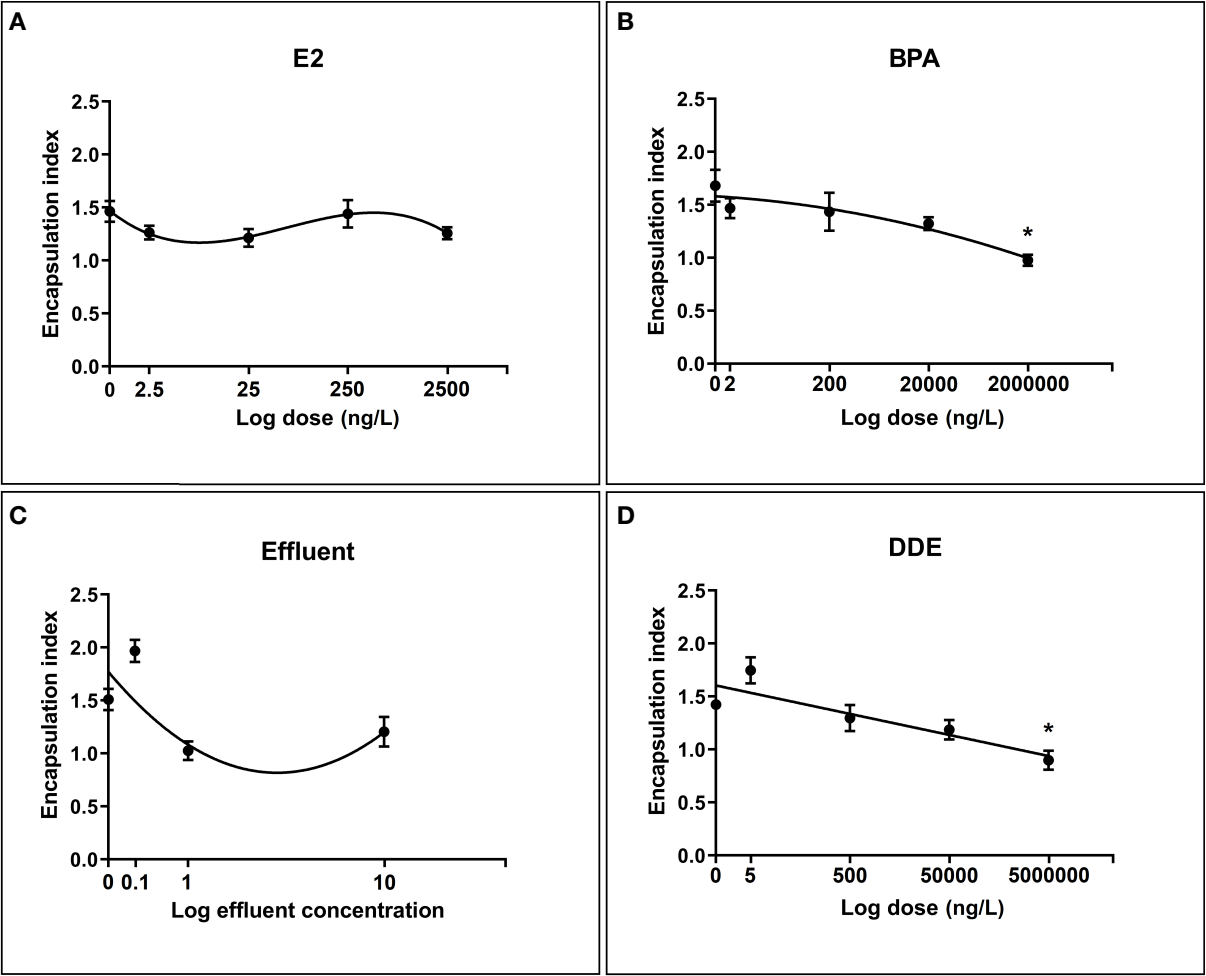
Regression relationships between log dose of test chemicals against encapsulation response toward *S. mansoni* sporocysts by *B glabrata* hemocytes exposed *in vitro*. Hemocyte response plotted as mean encapsulation index value ± SEM of three independent replicates. **(A)** Hemocyte encapsulation response to E2 exposure **(B)** Hemocyte encapsulation response to BPA exposure **(C)** Hemocyte encapsulation response to Effluent exposure **(D)** Hemocyte encapsulation response to p,p’-DDE exposure. Asterisks indicate significant differences to the respective control (p = < 0.05) according to Dunnett’s test for multiple comparisons.

All chemicals were best described in their relationship with the encapsulation index by a nonlinear dose-response curve. The relationship between encapsulation index and dose for BPA and the effluent extract was best modelled by a second order (quadratic) polynomial (y = a + bx + cx^2^) curve whereas E2 was best modelled by a third order (cubic) polynomial (Y = a + bx + cx^2^ + dx^3^). The dose-response relationship for p,p’-DDE was the most simplistic, being best described by a first order (straight line) polynomial model (y = b0 + b1x) (**Figure 3D**).

The results from the multiple comparisons test showed that only the top doses of BPA (2mg/L) and p,p’-DDE (5mg/L) were significantly different from their controls (**Figure 3**). Tukeys HSD multiple comparisons test was also performed to compare all chemicals and doses with each other. Numerous significant differences between combinations of different doses/chemicals were found, with 0.1x effluent appearing in the largest number of significant combinations. Within chemicals, aside from the differences shown by Dunnett’s test, a significant difference was found between 0.1x and both 1x and 10x for effluent and between 5ng/L and 5 mg/L for p,p’-DDE.

### Phagocytosis assay

**Figure 4:**
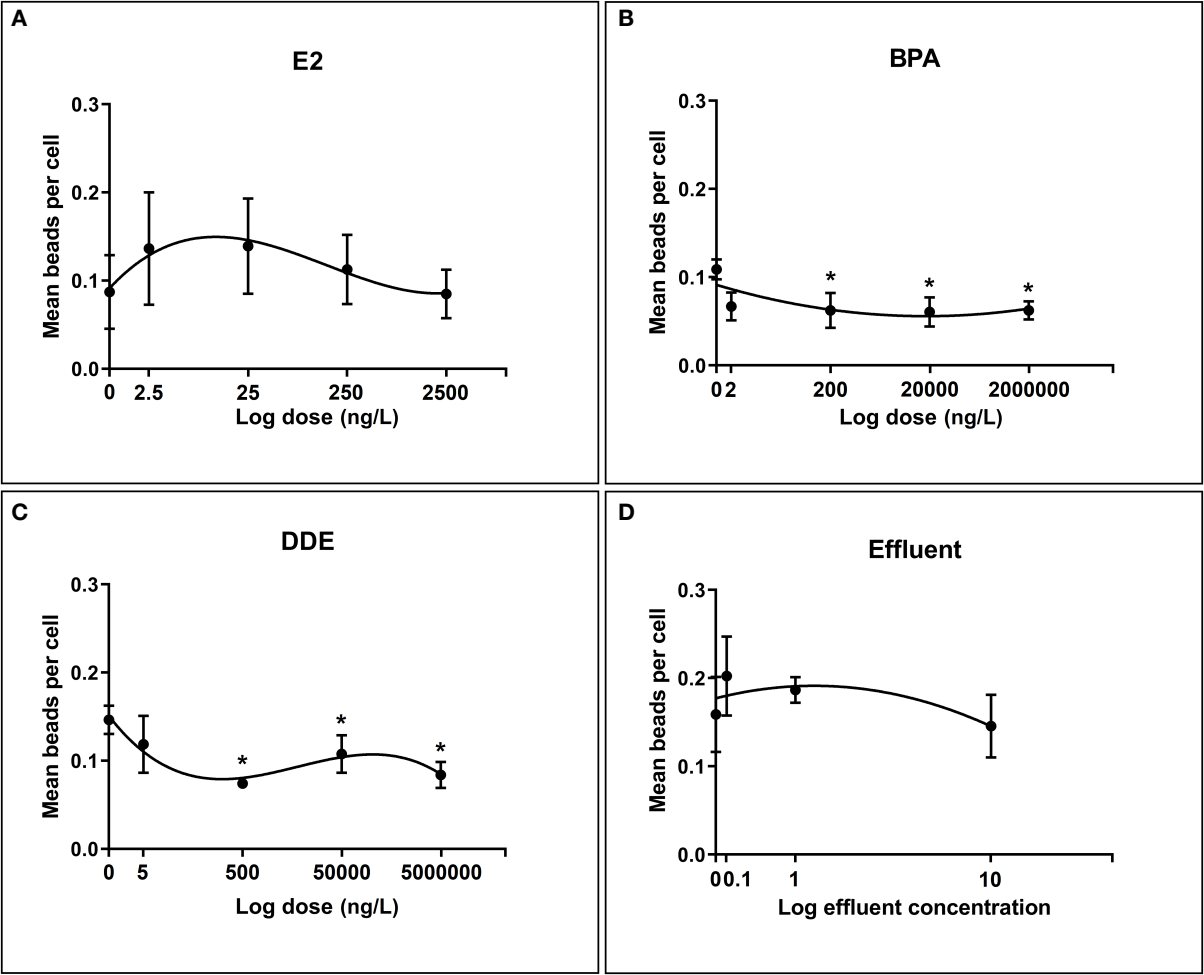
Regression relationships between log dose of test chemicals against phagocytosis of latex beads by *B glabrata* hemocytes exposed *in vitro*. Hemocyte response plotted as mean number of beads per cell ± SEM of four independent replicates. **(A)** Hemocyte phagocytosis response to E2 exposure **(B)** Hemocyte phagocytosis response to BPA exposure **(C)** Hemocyte phagocytosis response to Effluent exposure **(D)** Hemocyte phagocytosis response to p,p’-DDE exposure. Asterisks (*) indicate significant differences relative to the control (p = < 0.05) according to Dunnett’s test for multiple comparisons.

**Figure 4** illustrates the effect of chemicals and effluent extract on the ability of hemocytes to phagocytose beads. As with the encapsulation assays, all dose-response relationships were best fit by non-linear polynomials, with BPA and effluent following a second order (quadratic) curve and E2 and p,p’-DDE following a third order (cubic).

p,p’-DDE dose showed the strongest ability to predict the value of mean beads per cell explaining >59% of the variation (Adjusted R 2 = 0.5943). The next strongest relationship was for E2, where 32% of the variation in mean beads per cell was explained (Adjusted R 2 = 0.32), closely followed by BPA dose which explained 31% of the variation (Adjusted R 2 = 0.31). Effluent concentration showed a very weak relationship with phagocytosis response, accounting for just 6.8% of the variation in the mean number of beads per cell (Adjusted R 2 = 0.06851).

Results from the multiple comparisons test showed no significant difference from the control for E2 and the effluent extract. Indeed, variability in the level of phagocytosis in hemocyctes exposed to each dose of E2 varied widely as shown in **Figure 4**. For both BPA and p,p’-DDE the responses at the highest three doses (200ng/L, 20µg/L, 2mg/L and 500ng/L, 50µg/L and 5mg/L respectively) were found to be significantly lower than their respective controls, indicating a degree of inhibition in phagocytosis response at these doses.

### Motility assay

**Figure 5:**
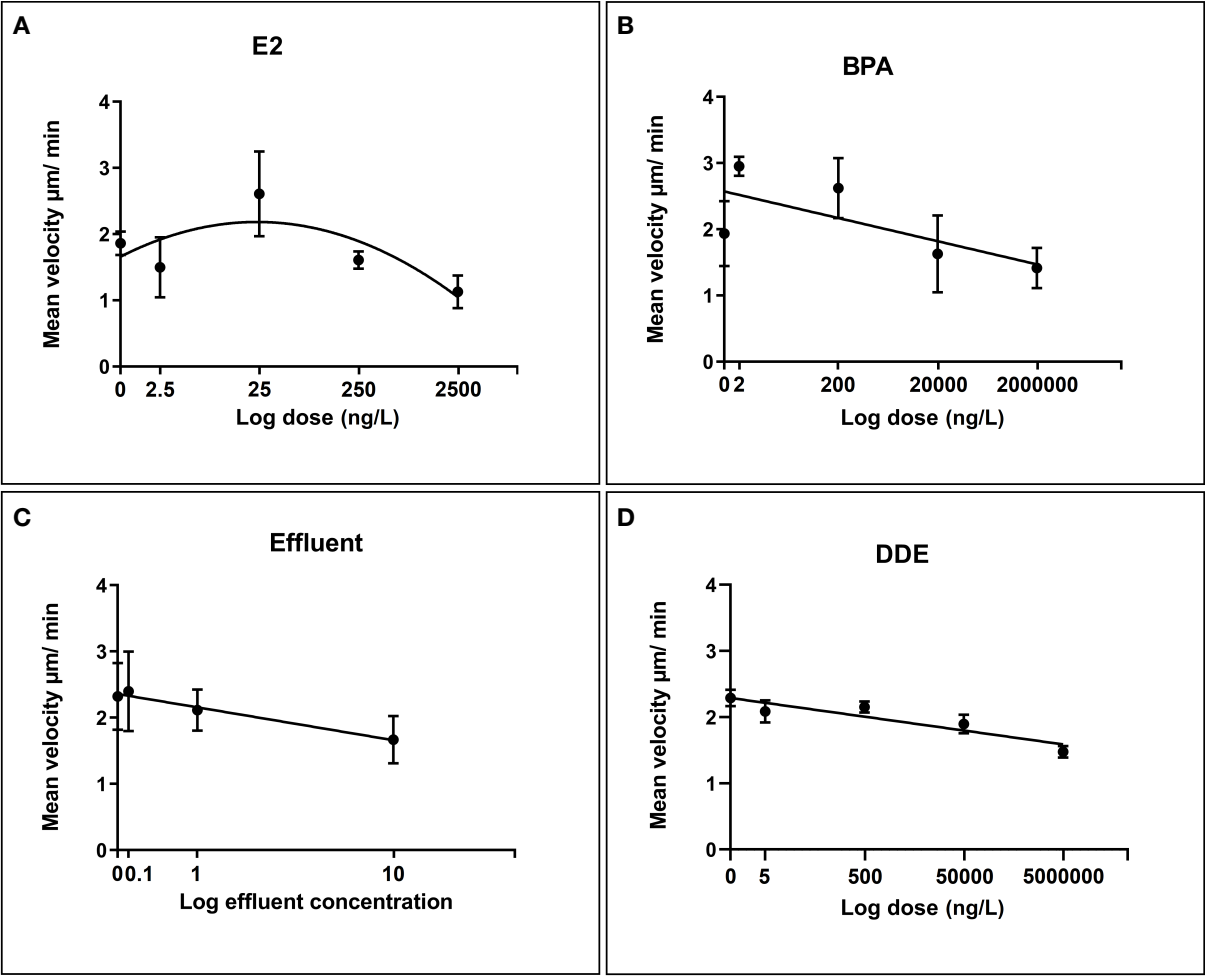
Regression relationships between log dose of test chemicals against *B glabrata* hemocyte motility *in vitro*. Data points for hemocyte motility plotted as mean velocity (µm/min) ± SEM of three independent replicates. **(A)** Hemocyte motility response to E2 exposure **(B)** Hemocyte motility response to BPA exposure **(C)** Hemocyte motility response to Effluent exposure **(D)** Hemocyte motility response to p,p’-DDE exposure.

With the exception of E2 (which was best modelled by a 2nd order polynomial), all chemicals followed a negative linear relationship (**Figure 5**). All relationships were relatively strong and suggested that the majority of variation in cell velocity was explainable by chemical dose. Effluent displayed the strongest relationship (Y = -0.6705*X + 2.359) with a very high r2 value of 0.9754 followed by p,p’-DDE (r2 = 0.8483, Y = -0.1046*X + 2.290) and E2 (Adjusted R2 of 0.8155). The relationship with BPA was somewhat weaker, but exposure still explained a considerable degree of the variation in cell velocity (r2 = 0.5122, Y = -0.1749*X + 2.572). BPA appeared to induce a degree of stimulation, relative to control, at the lowest dose but overall followed a negative linear relationship with increasing dose. In each case, diagnostic tests were satisfactory (i.e. normality, insignificant deviations from linearity in the case of BPA, effluent and p,p’-DDE).

## Discussion

The potential importance of chemically-induced changes to immune system function in molluscs inspired us to develop a novel toolkit of *in vitro* toxicity tests using mollusc hemocytes that broadly captures critical immune cell response behaviors. The ability of individual chemicals found in the aquatic environment, and an effluent extract from a typical UK tertiary-treated domestic sewage effluent, to influence hemocyte motility, phagocytosis and encapsulation has been assessed. Whilst developing the suite of immune tests we discovered that inter- and intra-variation in the responsiveness of snail hemocytes harvested from individual snails was relatively high compared to other types of cells (59–61). In *B. glabrata*, responses between individual snails can sometimes show considerable, and often unexplainable, variation. For example, the majority of cells from one animal may spread readily on contact with glass, while the majority of cells from another individual will remain rounded (44). Such variation introduces challenges when undertaking immunocompetence assays (62). In order to compensate for this variability we pooled hemolymph samples from several animals before splitting the sample into sub-samples on which *in vitro* tests are carried out. This approach is reported in a large number of studies (63, 64) and reduces the influence of unresponsive snails on test procedures, and provides a larger volume of hemolymph needed for sufficient biological replicates by most definitions (65).

Although the snail immune system can theoretically attack any intra-molluscan stage of the parasite, the primary response is usually directed toward the early sporocyst (66–68). In order to achieve a realistic *in vitro* representation of the snail immune response, it was necessary to use sporocysts, and this necessitated the *in vitro* transformation of collected miracidia. Sporocysts are defined as miracidia that have shed their ciliated plates and exhibit muscular squirming (69), and can be maintained *in vitro* for at least 14 days under basic conditions and potentially continuously, up to and including the production of cercariae, although this is considerably more difficult (69). For our purposes we were interested in early sporocysts around 6-hours after first contact with the medium as the ciliated plates have mostly shed and the syncytial tegument is formed. At this time the parasite is at the early stages of snail infection, and is most vulnerable to elimination by the snail immune system (70, 71). A variety of transformation media are reported in the literature but the most commonly used is the same CBSS used for hemocyte culture (71–73). Despite multiple tests using separate batches of CBSS, carried out at two different optimum pH values reported in the literature (7.2 and 7.4), it was not possible to achieve sufficient transformation of miracidia to sporocysts, let alone > 90% rates reported for CBSS (72, 74). Since CBSS worked very well for supporting hemocyte culture and untransformed miracidia, it is likely that this was due to differences in the parasite strain used, rather than the composition of the media. As an alternative transformation medium, we also tested Dulbecco’s modified Eagle’s medium (DMEM; Sigma-Aldrich, UK) supplemented with 10% fetal bovine serum and 2mM L-glutamine (69) because it is routinely used to culture adult worms. Indeed, we achieved transformation rates close to 100% using DMEM. Moreover, removal of the bovine serum did not alter the effectiveness of DMEM, which further simplified the method and reduced the cost. The collection jar system which combined multiple washing steps of the eggs, and the use of photo and chemotaxis behaviours of the hatched miracidia yielded a very clean preparation of sporocysts that was free of interfering mouse tissue. We also fixed sporocysts for use in the immune assays. Unlike internal features, the immunoreactivity of external features, including antigens, are well preserved by fixatives (75). Fixing parasites also prevented protein turnover and provided a more stable target (76) for repeated tests.

Given that the research was designed to detect alterations in hemocyte immune-responses and not direct cytotoxicity, it was important to test the effect of the chosen chemicals, across the chosen dose ranges, on hemocyte viability. Without establishing whether chemical doses result in reduced viability, we cannot establish to what extent an observed reduction in immune-response is a consequence of toxicity, as opposed to subtler and non-lethal changes in behavior and function of cells. None of the test chemicals showed any reduction in cell viability over the concentration range used according to the trypan blue exclusion assay. We also determined if chemically-induced alterations in the immune-responses of interest could be reliably measured in the absence of direct cytotoxicity. Sodium azide (NaN3) is known to be a potent cytochrome C inhibitor in many different cell-types from numerous species, including molluscan hemocytes (54). Inhibition of cytochrome C results in the inhibition of ATP-synthesis, ATP being the main source of energy for the majority of cellular functions (55). Cellular functions dependent on ATP, and thus shown to be inhibited by NaN3, include phagocytosis, motility and aggregation (54, 56). Studies involving molluscan hemocytes have reported NaN3 to considerably reduce phagocytosis without significant reduction in cell viability at concentrations of up to 2% (57, 58). Indeed, the response of each immune endpoint was strongly suppressed with increasing doses of NaN3, while hemocyte viability was minimally reduced across the same dose range. These results are in accordance with previous reports on the ability of NaN3 to suppress hemocyte function, without inducing significant loss in cell viability (54, 58).

There are presently no studies investigating *in vitro* encapsulation by invertebrate hemocytes in response to environmental contaminants. Despite this, a number of studies exist (the majority reported by Canesi and colleagues), on the effects of BPA and E2 on *in vitro* immune parameters in bivalve molluscs (mussels) due to their commercial importance. While their work does not investigate the encapsulation response, E2 was found to rapidly affect various immune parameters in mussel hemocytes through modulation of Mitogen-activated protein kinases (MAPK) via the activation of kinase cascades (34). Interestingly, since such functions are often well conserved, the same signaling pathways are believed to be important in regulating cell adherence and spreading in *B. glabrata*, which are two of the fundamental stages leading to encapsulation (77). From this we can speculate to a possible mechanism by which E2 may influence encapsulation. p,p’-DDE and the effluent extract appeared to display hormesis-like trends on encapsulation, characterized by low-dose stimulation and high-dose suppression. This phenomenon has been reported in a wide range of animal and plant species, and is also often associated with immune function dose-response relationships (78). Certain environmental pollutants do appear to exert a modest effect on *in vitro* sporocyst encapsulation, but the relationship is relatively complex. However, the aim was not to intentionally achieve a dramatic and stark response by using high doses which entirely disrupt the process. Concentrations of chemicals used in this study were chosen to ensure a degree of environmental relevance (based on water concentration alone), although we should be mindful that that concentrations of some chemicals in hemolymph, *in vivo*, could be significantly higher due to bioconcentration.

Quantifying phagocytosis in molluscan hemocytes is an innately challenging process resulting in considerable intra- and inter-assay variation regardless of the species for which they are used (79). This is potentially exacerbated by the fact that biological variation in primary cell cultures is typically considerably higher than in cell lines (80), and cell numbers from primary sources can be limited. Nevertheless, assays can be developed to study the process of phagocytosis in primary cells if their particular nature is considered and procedures are adapted accordingly. Given the nature of *B. gabrata* hemocytes, the measured intra and inter-pool CVs for phagocytosis of 6.6% and 22.3%, respectively, were considered satisfactory, and were consistent with a previous report using oyster hemocytes (81). A key advantages of imaging flow cytometry is its ability to fuse the sensitivity of microscope visualization with the high-throughput nature of standard flow cytometry *via* the collection of detailed images for every data point. To date there are no published examples of the application of imaging flow cytometry to mollusc hemocytes in any capacity. In fact, phagocytosis in *B. glabrata* has only been reported using standard flow cytometry by Bakry and colleagues (64). The majority of phagocytosis studies in molluscan hemocytes have quantified effects based on visual examination of cells under light microscopy. The phagocytosis dose-responses showed some moderate similarities to those of the encapsulation assays, possibly due to the similarities in the mechanisms that govern both responses. Phagocytosis responses following exposure to various doses of E2, BPA and the effluent extract were all best modelled by third-order polynomials, as was the case for encapsulation response. p,p’-DDE was the only chemical to show a markedly different shaped dose-response between phagocytosis and encapsulation (third-order polynomial vs. first-order respectively). The strength of the relationships were comparable, as were the results for Dunnett’s test when comparing against the control. As with the encapsulation assays, certain doses of BPA and p,p’-DDE produced responses that were significantly different from the control group. For the encapsulation assays only the highest doses of BPA and p,p’-DDE significantly inhibited the response compared to the control (**Figure 3**). However, all but the lowest dose of these chemicals (200ng-2mg; BPA and 500ng – 5mg; p,p’-DDE) significantly inhibited the phagocytosis response compared to the controls, suggesting that the phagocytosis assay is perhaps more sensitive than the encapsulation assay using these chemicals. An exception to the general similarities between the encapsulation and phagocytosis responses was observed with the effluent treatment, which showed a moderate to high coefficient of determination when predicting encapsulation but a very weak one when predicting phagocytosis. One possible explanation for the observed differences between these responses may be the choice of antigens used i.e. latex beads rather than sporocysts which are biological and have surface antigens. It has been shown that there may be differences in the responses of cells to different phagocytosis antigens under the influence of certain stressors (82).

Both E2 and BPA have generally been found to have a stimulatory effect on mollusc hemocyte phagocytosis at low (environmentally relevant) concentrations *in vitro* and inhibition at higher concentrations (83, 84). This non-monotonic dose response shape was somewhat evident in E2, with the first three doses appearing to have a higher mean response compared to the control, although the differences were not significant (**Figure 4**). It should be noted that in other reports the low dose stimulation often took longer (~6 hours) to occur than higher dose effects (83), and this time frame was longer than that used here. Comparisons with the literature are complicated by the fact that several studies use hemocytes collected from animals that have been injected with test chemical, after which the cells are quickly removed for analysis. In these cases, the quantity of chemical injected into the animals is known, but the precise level of systemic exposure to the cells is not (83, 84)

Only one other study exists describing the effects of xenobiotics on the motility of molluscan hemocytes (85). The *in vitro* motility of hemocytes from mussels collected at a polluted site was lower compared to a relatively non-polluted site (85). A direct comparison with our data is not possible due to differences in study designs, although we also found considerable reductions in hemocyte motility with increasing doses of our test chemicals. One exception to this was for E2, which showed a non-monotonic response in the middle of the dose range. In terms of mechanisms for the observed response, as with phagocytosis the ERK signaling pathway would again appear to represent a potential candidate since it is key in regulating cell motility and has also been shown to be negatively impacted in mollusc hemocytes by *in vitro* exposure to environmentally relevant doses of xenobiotics, including E2 and BPA (34, 86, 87).

To conclude, a novel suite of *in vitro* toxicity tests using mollusc hemocytes suggests that the innate immune system of *B. glabrata* is susceptible to the influence of aquatic pollutants. Although dose-response relationships were not always simple, there was a greater chance of suppression of immunity overall, even at environmentally-relevant concentrations. The assays we have developed can be used link changes to immune cell behavior in response to chemical exposures to molecular pathways, thereby enabling the development of Adverse Outcome Pathways (88) associated with vector borne diseases. Given the importance of *B. glabrata* as a vector for the transmission of Schistosomiasis to humans it will also be important to understand if early developmental exposure of *B. glabrata* to aquatic pollutants increases their susceptibility to parasitic infection, thereby potentially reinforcing transmission of the disease to humans.

## Data availability statement

The raw data supporting the conclusions of this article will be made available by the authors, without undue reservation.

## Author contributions

AL and ER conceived and designed the experiments. AL conducted the experiments. AL and ER analyzed the data. CJ and LN provided ongoing advice and technical support. ER wrote and revised the manuscript. All authors contributed to the article and approved the submitted version

## Funding

The studentship supporting this research was awarded by the UK Natural Environment Research Council (NERC).

## Acknowledgments

We are grateful to Dr. David Rollinson (Natural History Museum) for providing *B. glabrata* snails and to Drs. Nuha Mansour and Quentin Bickle (London School of Hygiene and Tropical Medicine) for providing parasite material. We thank the UK Natural Environment Research Council (NERC) for funding.

## Conflict of interest

The authors declare that the research was conducted in the absence of any commercial or financial relationships that could be construed as a potential conflict of interest.

## Publisher’s note

All claims expressed in this article are solely those of the authors and do not necessarily represent those of their affiliated organizations, or those of the publisher, the editors and the reviewers. Any product that may be evaluated in this article, or claim that may be made by its manufacturer, is not guaranteed or endorsed by the publisher.
