# Supplementary material for: Common aquatic pollutants modify hemocyte immune responses in Biomphalaria glabrata
Source: Front Immunol. 2022 Sep 8;13:839746. doi: 10.3389/fimmu.2022.839746 (PMC9493456; doi:10.3389/fimmu.2022.839746)
Supplement: Supplementary file 1 [file DataSheet_1.pdf]

Supplementary Material

Egg hatching and collection of Miracidia

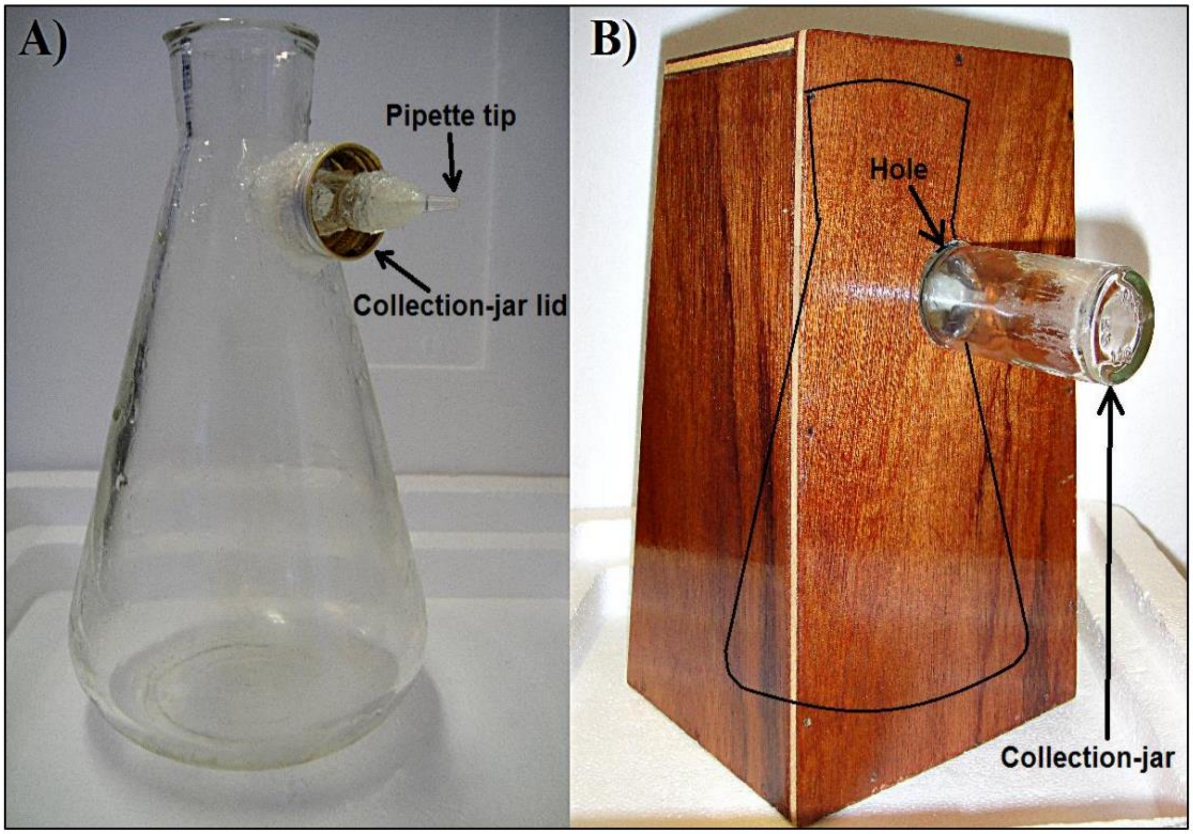

Supplementary Figure 1. Photographs of my miracidia collection device. A) 500ml side-arm flask for collection of liver homogenate. The side-arm was adapted with the addition of a bottle cap and p20 pipette tip B) Wooden box constructed to house flask, the collection bottle can be seen attached and extruding so that light is only exposed to the collection jar where miracidia will pool themselves.

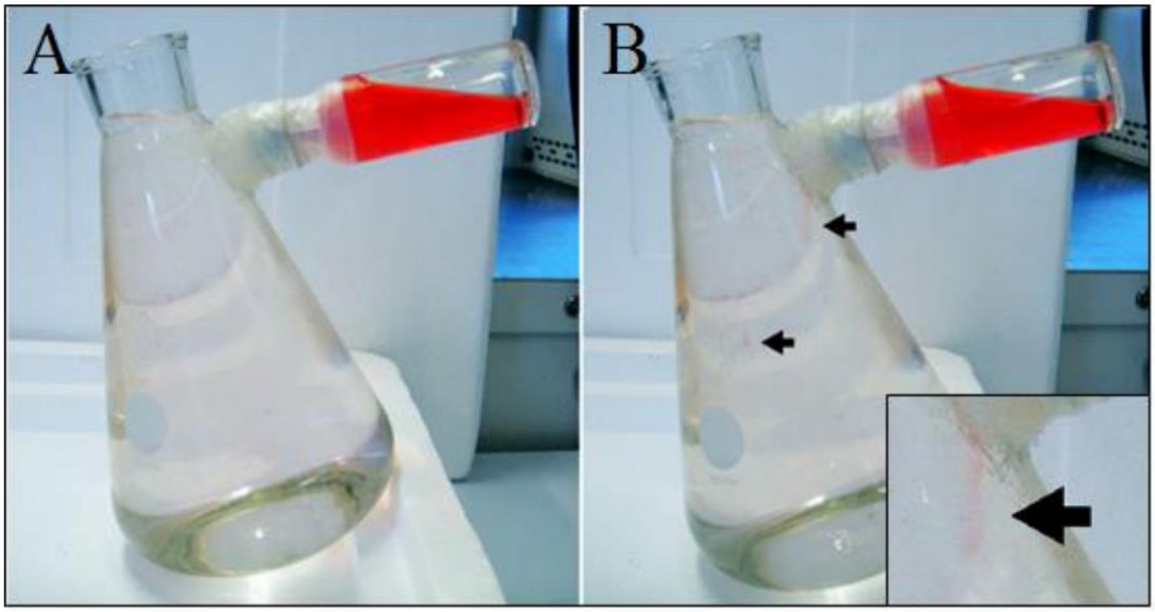

Supplementary Figure 2. Demonstration of the chemical gradient formed between the miracidia collection chamber and the main flask. Red dye was used to allow observation of the gradient, A) after 0 hours B) after 1 hour. Arrows indicate the dye trail – the enlarged section shows the trail in more detail.

Encapsulation Assay

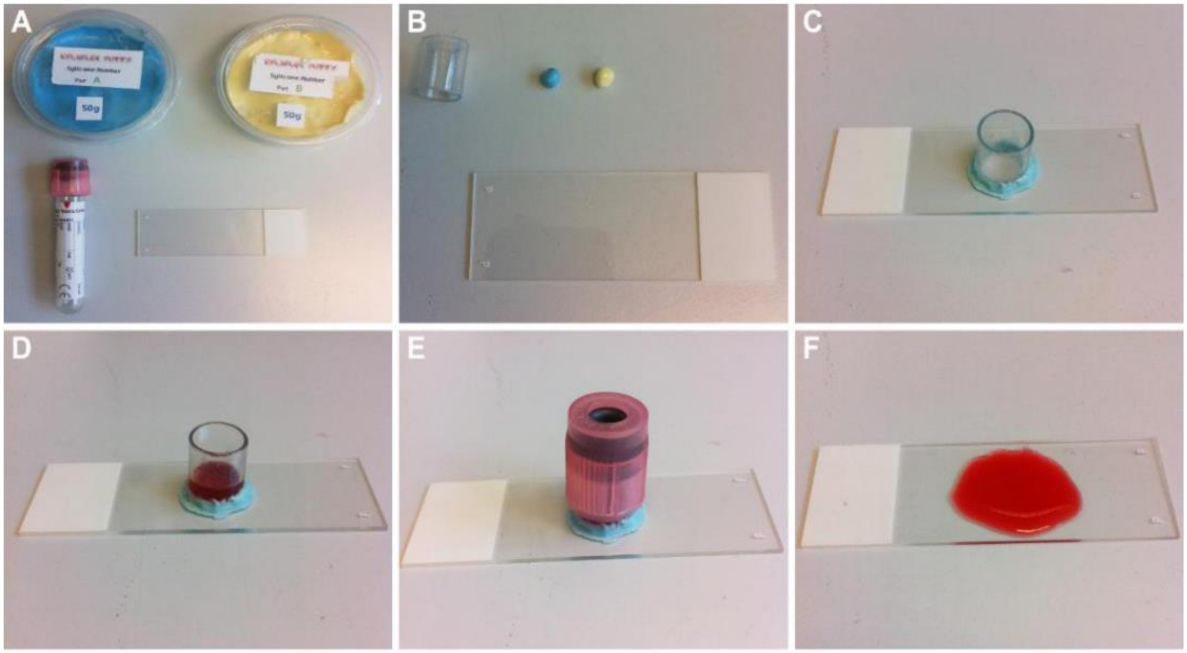

Supplementary Figure 3. Development of purpose-built chamber-slide for use in the *in vitro* encapsulation assay. A) The individual components used to construct the slides; silicone putty consisting of two parts, Vacutainer tube and glass microscope slide B) The individual components prepared for set-up; the Vacutainer tube cut to size and the appropriate amount of the two putty parts C) Chamber slide prepared for use, with the putty parts combined and hardened D) Chamber filled with hemolymph E) Vacutainer top added back on to the tube section to prevent desiccation/evaporation F) The resulting state of the hemolymph sample after removal of the tube chamber and putty; no residual putty left behind, the sample is now ready for application of a coverslip for observations.

$$\textit{Assay encapsulation index value} = \frac{\textit{Mean score of all sporocysts}}{\textit{Total sporocysts counted}}$$

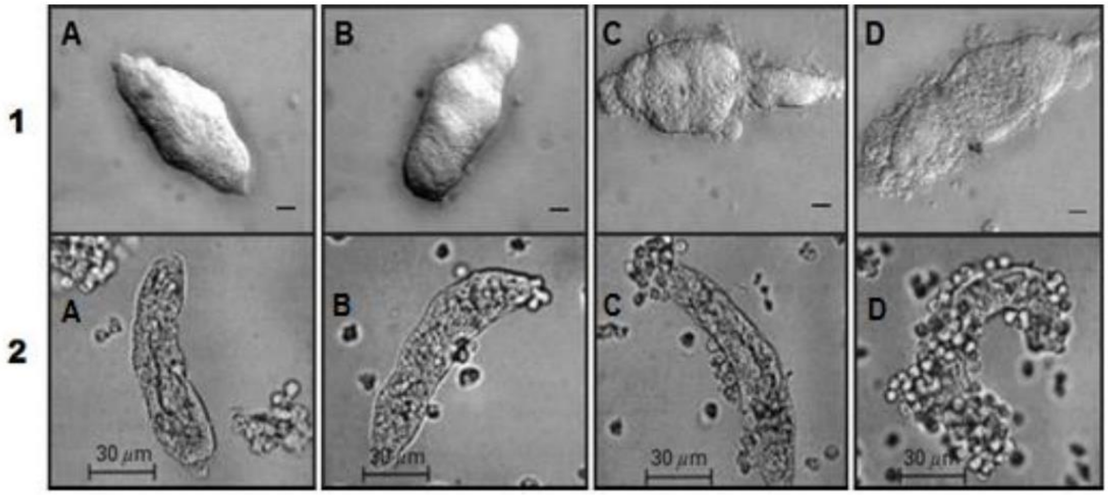

Supplementary Figure 4. Photographic examples of sporocyst encapsulation index from two different studies. Row 1, A-D; encapsulation index values 0-3 according to Martins-Souza et al., (2011). Row 2, A-D; encapsulation index values 1-4 according to Castilllo and Yoshino (2002)

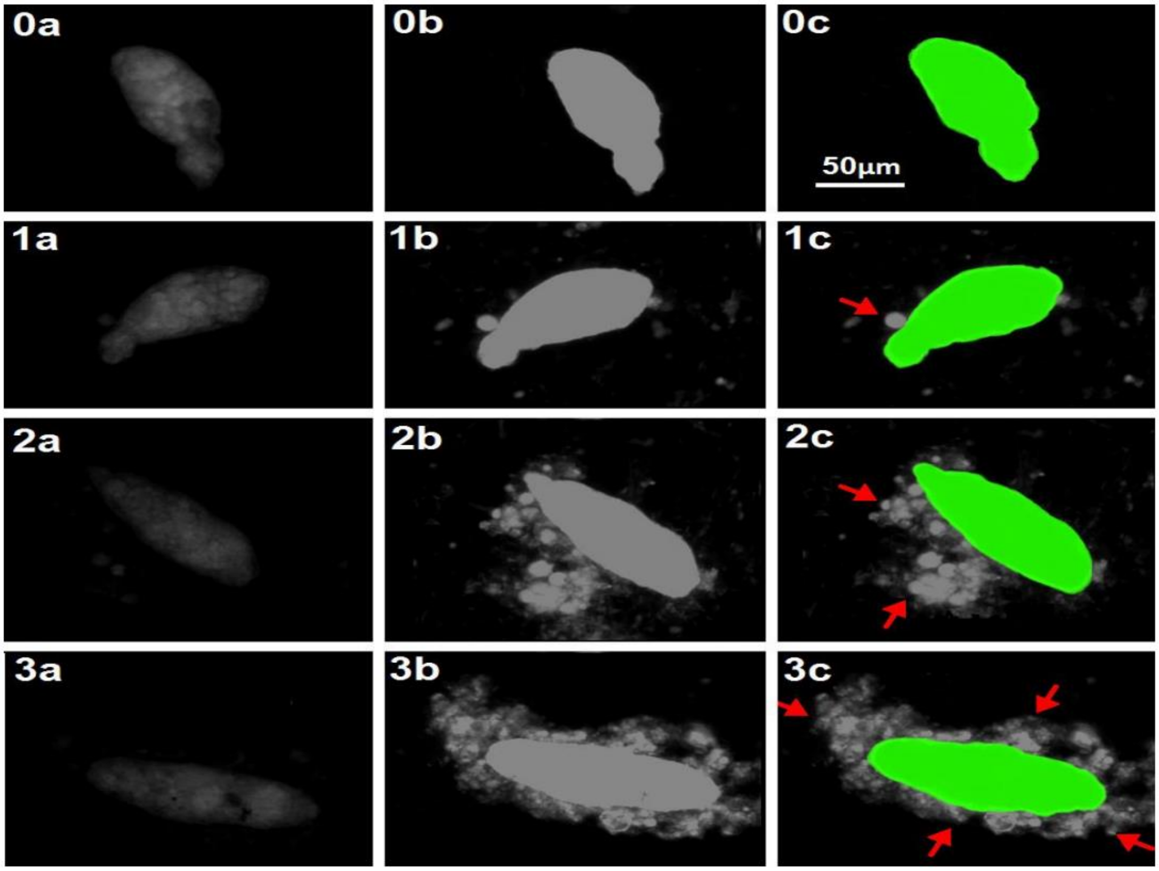

Supplementary Figure 5. Example of fluorescence microscopy used in the scoring index of my encapsulation assays. A) Example of how fluorescent sporocysts can be identified even when encapsulating B) Shows that adjustment of exposure and contrast allows observation of a 2D image of the sporocyst and the attached cells C) Shows the sporocysts after being masked to better distinguish the parasite from the surrounding cells. Arrows indicate attached hemocytes

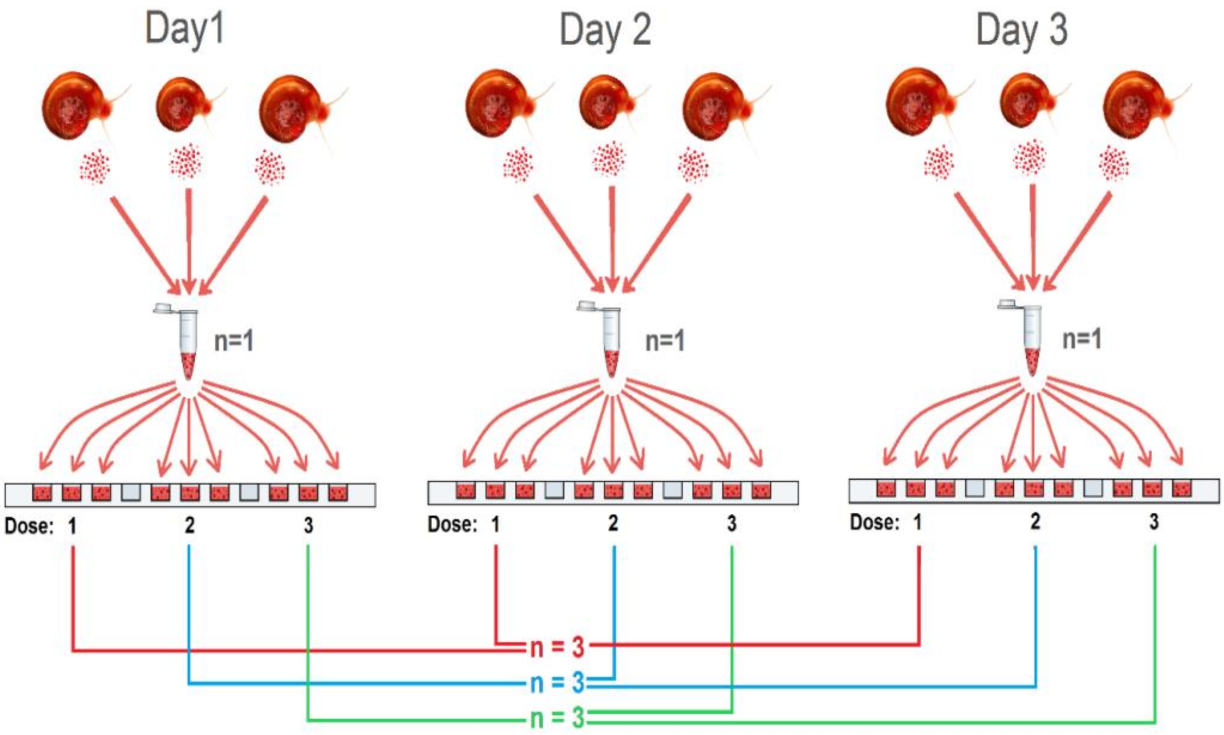

Supplementary Figure 6. Diagrammatic example illustrating the process whereby technical and experimental units are generated in the various immune assays. On the day of an assay, hemolymph from individual snails (typically 8/day) were pooled, mixed and then allocated to a specific treatment to form a single experimental unit. This process was repeated on a different days, with different snails (it is a destructive process and repeated sampling is not possible), to produce a number of independent replicates for each experimental unit (chemical and dose). In this schematic representation, three snails and their converging arrows show how hemocytes from each group of snails was pooled into a single tube on three consecutive days. The subsequent nine arrows indicate how samples of pooled hemolymph were allocated to individual wells (with dose 1-3 representing different treatments e.g. control and the various chemical doses). Lines of the same colour (red, blue or green) show how wells contribute to the experimental units for each dose (in this case n=3, taken as an average of the three technical replicates). The approach allows a level of consistency in the cell population used to test each chemical, by ensuring that the effects of biological variability are distributed more evenly across each dose.
